# The Relationship Between Feelings of Emptiness and Self-Harm Among Thai Patients Exhibiting Borderline Personality Disorder Symptoms: The Mediating Role of the Inner Strengths

**DOI:** 10.3390/medicina60111776

**Published:** 2024-10-30

**Authors:** Piangdao Sripunya, Tinakon Wongpakaran, Nahathai Wongpakaran

**Affiliations:** Department of Psychiatry, Faculty of Medicine, Chiang Mai University, Chiang Mai 50200, Thailand; piangdao.sr@cmu.ac.th (P.S.); nahathai.wongpakaran@cmu.ac.th (N.W.)

**Keywords:** emotions, self-injurious behavior, borderline personality disorder, Buddhism, meditation

## Abstract

*Background and Objectives*: Fifty percent of individuals with borderline personality disorder (BPD) experience self-harm. One of the crucial factors related to self-harm is feelings of emptiness. While inner strengths, such as the Five Precepts, meditation, and equanimity, have been identified as potential buffers against negative mental health outcomes in BPD, their role in mediating the relationship between feelings of emptiness and self-harm is not well-documented. This study aimed to explore how these inner strengths mediate the relationship between feelings of emptiness and self-harm in individuals exhibiting BPD symptoms. *Materials and Methods*: A total of 302 Thai participants exhibiting BPD symptoms completed several assessments: the SCID-II Personality Disorder Questionnaire for BPD to assess feelings of emptiness and self-harm, the Inner-Strength-Based Inventory (i-SBI) to evaluate the Five Precepts, meditation, and equanimity, and the Outcome Inventory Depression (OI-Depression) to assess depression. Mean and standard deviation were used for continuous variables, such as age and OI-Depression. A *t*-test assessed mean differences in continuous variables between the self-harm group and the non-self-harm group. Chi-square tests examined differences in categorical variables with three or more levels, such as education. Pearson’s correlation and linear regression analyzed relationships between continuous variables, including i-SBI and OI-Depression scores. Mediation analysis was performed using IBM SPSS and AMOS, with self-harm as the outcome variable, feelings of emptiness as the predictor, and inner strengths as mediators. *Results*: The participants had a mean age of 36.56, with 65.4% being female. The analysis showed that the Five Precepts, meditation, and equanimity significantly mediated the relationship between feelings of emptiness and self-harm, with a standardized coefficient of β = 0.534 (95% CI = 0.417 to 0.647, *p* < 0.001). The indirect effect of feelings of emptiness through these inner strengths was significant (β = 0.034, 95% CI = 0.009 to 0.075, *p* = 0.005). The mediation model explained 38% of the variance in self-harm with a 3% increase, albeit small but significant. *Conclusions*: This study highlights that inner strengths negatively mediate the relationship between feelings of emptiness and self-harm, indicating that as these inner strengths increase, the direct impact of feelings of emptiness on self-harm decreases. These findings suggest that targeting inner strengths as protective factors could be a valuable strategy in developing interventions aimed at reducing self-harm by addressing the underlying emotional challenges associated with BPD.

## 1. Introduction

Self-harm, including non-suicidal self-injury (NSSI), or self-mutilation, involves the deliberate destruction of body tissue without suicidal intent and is common in borderline personality disorder (BPD). BPD is characterized by unstable interpersonal relationships, fear of abandonment, difficulties in emotion regulation, feelings of emptiness, chronic dysphoria, depression, impulsivity, and heightened risk-taking behaviors. BPD can be understood as stemming from difficulties in early dyadic regulation with primary caregivers, such as early insecure attachment, which results in under-regulation of emotions and fear of abandonment [1,2]. In Thailand, the prevalence of BPD among Thai students has been reported as 6.4% [3]. International epidemiological studies estimate the prevalence of BPD in the general population to be between 0.7% and 2.7%. In the U.S., the prevalence among adolescents is approximately 1%, with a cumulative rate of 3% by age 22 [4]. BPD often involves recurring self-injurious or suicidal behavior [5]. Self-harm occurs in 50% to 80% of BPD cases, primarily through methods such as cutting. While self-harm may temporarily relieve distress, elicit care from others, express emotions symbolically, or help patients emerge from dissociation and feel more alive, it increases the risk of suicide, depression, feelings of hopelessness, worsening emotional instability, aggression, and suicidal thoughts [6,7,8,9].

Another symptom in BPD is feelings of emptiness, strongly linked to greater borderline pathology [10] and associated with suicidal and self-injurious behaviors [11]. This complex emotional state includes feelings of aloneness, numbness, a deep sense of personal unfulfillment, and a lack of purpose. It often manifests as a sense of living mechanically, disconnected from others [12], and responses to feelings of emptiness vary [13]. Feelings of emptiness reflect pathological affect and significant psychiatric distress [14], contributing to functional impairment, such as lower social functioning [15,16]. Feelings of emptiness have been linked to self-harm, NSSI, and suicidality [17], with higher levels predicting increased suicide urges [11]. Evidence has shown that among the individuals exhibiting BPD symptoms, past suicidality, impulsivity, chronic emptiness, and identity disturbance were each significantly and positively associated with a lifetime history of self-harm [18]. Deficits in mindfulness—difficulty being aware, attentive, and accepting of ongoing experiences—further contribute to the relationship between BPD and self-harm, as well as overall harmful, dysregulated behaviors and suicidal ideation [19,20,21,22]. Mindfulness is negatively correlated with self-harm and mediates the relationship between depressive symptoms and self-harm [23].

Many studies show that meditation or mindfulness reduces psychological symptoms in BPD, improving emotional regulation and behavior [24,25,26]. Mindfulness enhances awareness of feelings of emptiness, helps identify related emotions, and promotes reflection on dysfunctional behaviors [27]. However, research on mediators between feelings of emptiness and self-harm is limited. One study found that mindfulness and emotion dysregulation mediated this relationship, with chronic feelings of emptiness indirectly affecting self-harm through mindfulness strategies [20,28]. Another related study demonstrated that perseverance and meditation were significant moderators for BPD symptoms and depression [29].

As the majority of Thais are Buddhists, meditation—part of the Ten Perfections—is a significant practice for increasing inner strength [30]. The Five Precepts—abstaining from killing, stealing, sexual misconduct, lying, and the consumption of alcohol and other intoxicants—are essential practices alongside meditation and promote mental health [31,32]. However, their effects on BPD remain underexplored. In addition to meditation and the Five Precepts, there are truthfulness, perseverance, wisdom, generosity, patience, endurance, equanimity, determination, and loving-kindness, all of which are related to positive mental health and serve as protective factors against mental health problems [30,33].

Equanimity, a state of psychological stability and composure that remains undisturbed by emotions, pain, or other circumstances, represents a balanced emotional reaction to stimuli and a tolerant, nonjudgmental attitude [34]. It is essential for well-being and often accompanies mindfulness, with meditation techniques available to cultivate it [35]. In Buddhism, equanimity is a virtuous state of mind rooted in freedom [36]. Studies show it improves emotional regulation [37,38], reduces anxiety and depression [39], and moderates the relationship between perceived stress, neuroticism, and depressive symptoms [40]. Equanimity also mediates the impact of perceived social isolation on psychological distress, providing protection against its negative effects [41]. Since individuals with BPD struggle with emotional regulation, practicing the Five Precepts, meditation, and cultivating equanimity may theoretically help reduce BPD symptoms, including self-harm, by promoting self-control and emotional regulation. The objective of this study was to investigate these particular strengths—namely, the Five Precepts, meditation, and equanimity—to determine whether they mediate the relationship between feelings of emptiness and self-harm among Thai patients exhibiting BPD symptoms attending a psychotherapy clinic. The authors hypothesized that a high level of adherence to the Five Precepts, meditation, and equanimity would mitigate the effects of feelings of emptiness on self-harm. Since depression is associated with both BPD and self-harm, it was controlled to avoid confounding.

## 2. Materials and Methods

### 2.1. Study Design and Sample Size Calculation

This study employed a retrospective cross-sectional design, using data from the psychotherapy clinic database at Maharaj Nakorn Chiang Mai Hospital, Faculty of Medicine, Chiang Mai University. The participants were either self-referred or referred by other psychiatrists for combined psychotherapy treatment. Intake interviews were conducted by a psychiatrist at the clinic to assess whether the patients would be accepted for psychotherapy. If accepted, further steps were completed, including the completion of a self-report questionnaire related to psychotherapy, obtaining informed consent, and developing a psychotherapy treatment plan. Data were gathered from November 2011 to August 2022, covering a study period of 10 years and 9 months. Power and sample size estimation was carried out for the mediation analysis model [42]. Two parallel mediators with a correlation coefficient of variables (Power (beta) = 0.8 (80%), Correlation coefficient (*r*) of emptiness, and self-harm = 0.39) yielded a sample size of 96, target power = 0.8 (95% CI = 0.77–0.83). Ethics review and approval were obtained from the Faculty of Medicine, Chiang Mai University, Thailand (study code: PSY-2566-0414; date of approval: 26 December 2023).

### 2.2. Participants and Setting

Data from all 337 participants stored in the database were used. These participants were patients who attended the psychotherapy clinic from November 2011 to August 2022. Participants were required to be at least 20 years old, able to speak Thai, of any gender, and diagnosed with a mental disorder or given a psychiatric diagnosis, including DSM personality disorder, and to have completed self-reports on the relevant measures. The exclusion criteria included individuals without a mental disorder or psychiatric diagnosis, without a DSM personality disorder diagnosis, or with incomplete data in their medical records. All participants signed written informed consent for psychotherapy. A total of 35 participants had incomplete reports; therefore, 302 participants with borderline personality disorder (BPD) symptoms were included in the final analysis. The attending psychiatrists determined the diagnoses using both the Structured Clinical Interview for DSM-IV Axis II Personality Disorders Questionnaire: Borderline Personality Disorder (SCID-II-PQ: BPD) and clinical interviews.

### 2.3. Measurements

#### 2.3.1. Medical Records for Sociodemographic Data

Sociodemographic data such as age, sex, and psychiatric diagnoses were extracted from medical records.

#### 2.3.2. The Structural Clinical Interview for DSM-IV Axis II Personality Disorders Questionnaire: Borderline Personality Disorder (SCID-II-PQ: BPD)

The SCID-II-PQ: BPD is a tool used in clinical settings to assess the presence of borderline personality disorder based on the Diagnostic and Statistical Manual of Mental Disorders, Fourth Edition (DSM-IV) and Fifth Edition (DSM-5). Specifically, the SCID-II-PQ is a self-report questionnaire that helps gather information about participants’ symptoms and behaviors related to personality disorders. First and colleagues developed it to complement diagnostic interviews conducted by psychiatrists. Following standard protocols for diagnosis with the SCID-II tool, the research assistant administered the SCID-II-PQ. The SCID-II-PQ: BPD includes nine items that cover crucial diagnostic criteria for BPD, such as instability in relationships, self-image, and emotions, as well as impulsivity and self-harm behaviors. Participants responded with ‘Yes’ or ‘No’ based on their compatibility with the items, and the results indicated the corresponding personality subtype they had [43].

This study used the Thai version of the scale, translated and validated by Wongpakaran and colleagues. The overall interrater reliability demonstrated good consistency across all studies, with the Kappa value between the first and second raters regarding the diagnosis of BPD being 0.84. Cronbach’s alpha for BPD was 0.90, indicating acceptable to excellent internal consistency [44].

#### 2.3.3. Inner-Strength-Based Inventory (i-SBI)

The i-SBI measures ten positive behavioral characteristics inspired by the Buddhist ten perfections: generosity, morality, mindfulness, meditation, wisdom, perseverance, patience and endurance, truthfulness, determination, loving-kindness, and equanimity. Each characteristic is assessed through multiple-choice responses on a 5-point scale. The i-SBI scores range from 5 to 50, with higher scores reflecting stronger characteristics. Each subscale can also be used independently. The Thai version of the i-SBI shows good psychometric properties, with person separation at 2.45, corresponding to good reliability (r = 0.86), and item reliability at 0.99 (>0.80). Cronbach’s alpha values were 0.74 in adolescents, 0.71 in adults, and 0.71 in older adults. For this analysis, we used items related to the Five Precepts (scoring: 1–2 low, 3 average, 4–5 high), meditation (scoring: 1 low, 2–3 average, 4–5 high), and equanimity (scoring: 1–2 low, 3–4 average, 5 high) [30].

#### 2.3.4. Outcome Inventory-Depression (OI-Depression)

OI-Depression is a component of the Outcome Inventory-21 (OI-21), which consists of 21 questions with subscales for anxiety, depression, interpersonal difficulties, and somatization. All items are rated on a 5-point Likert scale, with values of 1 (never), 2 (rarely), 3 (sometimes), 4 (frequently), and 5 (almost always). The total scores range from 0 to 48. OI-21 scores were grouped into three categories: “low” for scores below the 25th percentile, “average” for scores ranging from the 25th to the 75th percentile, and “high” for scores above the 75th percentile. Higher scores reflect higher levels of psychopathology. OI-Depression items 2, 5, 14, 18, and 21 were used in this analysis. The validity and reliability of the OI-Depression scale were well-established, with a sensitivity of 86.15%, specificity of 80.25%, positive predictive value of 78.30%, and negative predictive value of 87.50%. The area under the ROC curve was 0.89, indicating good diagnostic performance. Cronbach’s alpha for depression was 0.82 [45].

### 2.4. Statistical Analysis

Descriptive statistics were used to analyze demographic data such as age, gender, education, and marital status from medical records. Mean and standard deviation were used for continuous variables such as age and OI-Depression scores. A *t*-test assessed mean differences between the self-harm and non-self-harm groups, with self-harm identified through self-harm items in the SCID-II for Borderline PD. Chi-square tests examined differences in categorical variables with three or more levels, such as education. Correlation and linear regression analyses were conducted on continuous variables, while Pearson’s correlation was used to explore relationships between the i-SBI and OI-Depression scores.

The sample was assessed for normal distribution using AMOS. Skewness and kurtosis values indicated normality when they fell within the acceptable range of ±3. A multivariate normality test was conducted using Mahalanobis distance squared for the mediation analysis. Common thresholds for identifying outliers are based on the Chi-squared distribution; specifically, a Mahalanobis distance corresponding to a *p*-value of 0.001 is often utilized to detect potential outliers in datasets with two variables.

Mediation analyses examined significant correlations between feelings of emptiness in BPD symptoms (X) and the Five Precepts, meditation, and equanimity (M1–M3), with self-harm as the outcome variable (Y). Direct and indirect effects of feelings of emptiness on self-harm were assessed for significance. A 5000-resampling or bootstrapping method was applied to ensure more accurate results. Standardized regression coefficients (β) and *p*-values were reported for direct effect coefficients, while bootstrap confidence intervals were reported for indirect effects. Confidence intervals excluding zero indicated statistical significance. All analyses were performed using IBM SPSS (Version 25.0) and AMOS (Version 24) (IBM Corp., Armonk, NY, USA). The level of significance for all analyses was set at *p* < 0.05.

## 3. Results

**Figure 1:**
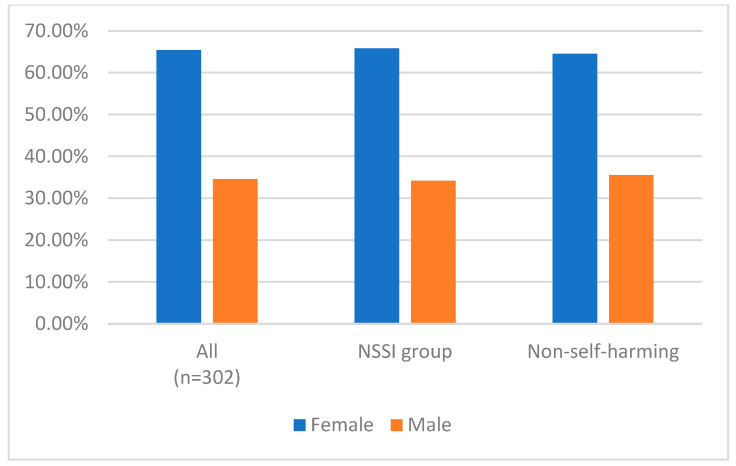
The distribution of sex in each group.

Among all participants, most were female. The mean and standard deviation of the scores of the measurements are shown in Table 1. Significant differences in age, feelings of emptiness, perceived stress, and OI-Depression were found between the self-harm and non-self-harm groups. Feelings of emptiness were higher in the self-harm group. Figure 1 shows the distribution of sex in each group.

Table 2 shows the correlation coefficients between each pair of variables. The correlations were as follows: emptiness and self-harm (0.591), OI-Depression was positively correlated with emptiness and self-harm (all *p* < 0.01).

Feelings of emptiness were negatively correlated with the Five Precepts, meditation, and equanimity (*p* < 0.01). Similarly, self-harm was negatively associated with the Five Precepts, meditation, and equanimity (*p* < 0.01).

The standardized regression coefficient for the effect of feelings of emptiness on self-harm was β = 0.591 (95% CI = 0.543 to 0.742, *p* < 0.001). OI-Depression was used to control for confounding factors related to depression. Table 3 shows that when controlling for age and OI-Depression, the regression coefficient for the effect of feelings of emptiness on self-harm was reduced to β = 0.565 (95% CI = 0.507 to 0.721, *p* < 0.001). Even with these controls, the relationship between feelings of emptiness and self-harm remained significant. This model explained 35% of the variance in self-harm.

Based on the correlations among variables, we hypothesized a mediation model. The Five Precepts, meditation, and equanimity, were entered as mediators in the relationship between feelings of emptiness and self-harm, with self-harm as the dependent variable.

Before conducting the mediation analysis, we assessed the normal distribution of the data. The results indicated that the skewness and kurtosis values ranged from −1.5 to 1.1, suggesting normality. Additionally, the Mahalanobis distance squared results showed that no *p*-values were equal to or less than 0.001, indicating no significant outliers were detected in the dataset.

The analysis assessed the effects of feelings of emptiness on self-harm both directly and indirectly, through the Five Precepts, meditation, and equanimity. The mediation model controlled for age and OI-Depression. The standardized total effect of feelings of emptiness on self-harm was β = 0.568 (95% CI = 0.453 to 0.675, *p* < 0.001).

**Figure 2:**
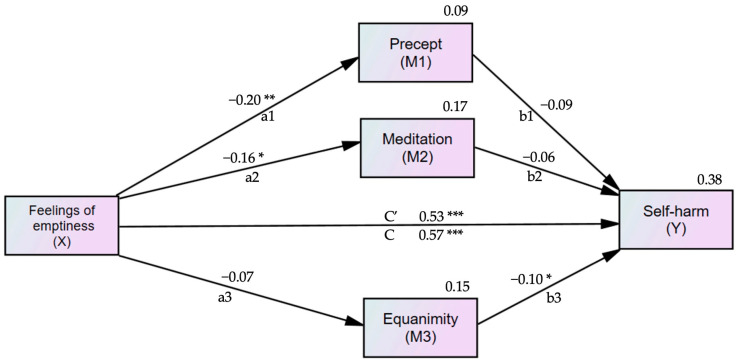
Mediation model of feelings of emptiness in patients with borderline personality disorder symptoms (BPD symptoms), with the Five Precepts, meditation, equanimity, and self-harm, adjusted for age and OI-Depression. X = Predictor; M1, M2, M3 = Mediator; Y = Outcome; a1, a2, a3, b1, b2, b3, c, c′ = Path coefficients; c = Total direct effect of feelings of emptiness on self-harm; c′ = Direct effect of feelings of emptiness on self-harm mediated by the Five Precepts, meditation, and equanimity. The value for self-harm (Y) is the R-square. Self-harm = non-suicidal self-injury. * *p* < 0.05, ** *p* < 0.01, *** *p* < 0.001.

Figure 2 shows the standardized estimation coefficients for the direct effects of feelings of emptiness on self-harm. The direct impact of feelings of emptiness was reduced from β = 0.565 (95% CI = 0.507 to 0.721, *p* < 0.001) to β = 0.534 (95% CI = 0.417 to 0.647, *p* < 0.001) when controlling for the mediators. The predictors in the mediation model explained 38% of the variance in self-harm.

Table 4 shows the direct effects of feelings of emptiness on the Five Precepts, meditation, equanimity, and self-harm. It also presents the direct effects of the Five Precepts, meditation, and equanimity on self-harm. The indirect effect of feelings of emptiness on self-harm was β = 0.034 (95% CI = 0.009 to 0.075, *p* = 0.005). For each variable of inner strength, meditation was correlated with the Five Precepts (*r* = 0.35) and equanimity (*r* = 0.10).

## 4. Discussion

This study is the first to investigate how the Five Precepts, meditation, and equanimity mediate the relationship between feelings of emptiness and self-harm. The findings highlight a significant indirect effect of feelings of emptiness on self-harm through these inner strengths among individuals with BPD symptoms. The negative mediating role suggests that as these inner strengths increase, the direct impact of feelings of emptiness on self-harm decreases. In other words, stronger inner resources—like adherence to the Five Precepts, regular meditation, and equanimity—reduce the connection between feeling empty and engaging in self-harm. This suggests that these inner strengths may act as protective factors, reducing the likelihood that someone feeling emptiness will resort to self-harm as a coping mechanism. In this context, inner strengths help buffer or diminish the impact of emotional emptiness on the likelihood of engaging in self-harm.

The prevalence of BPD among Thai students was 6.4%, higher than nationwide epidemiological estimates for the general population, which range from 0.7% to 2.7%. Feelings of emptiness, strongly associated with greater borderline pathology, are also linked to self-harm [10,18]. The relationship between feelings of emptiness and self-harm found in this study aligns with previous research. Deficits in mindfulness further contribute to the relationship between BPD and self-harm [19,20,21,22]. Meditation and adherence to the Five Precepts—abstaining from killing, stealing, sexual misconduct, lying, and the consumption of alcohol and other intoxicants—may help individuals become more aware, control negative experiences, reduce psychological symptoms in BPD, and improve emotional regulation and behavior [24,25,26]. Although no direct study can be referenced, related studies have demonstrated the role of meditation and the Five Precepts in the relationship between secure attachment and resilience [32]. Additionally, the Five Precepts are associated with higher levels of happiness [31], and negatively related to aggression, neuroticism, and sensation-seeking [33]. Equanimity—a state of psychological stability and composure undisturbed by emotions, pain, or other circumstances—represents a balanced emotional reaction and a tolerant, nonjudgmental attitude [34]. It has been linked to decreased anxiety and depression and improved emotional regulation and mitigates the effects of perceived stress on depression [37,38,39,40]. These combined inner strengths demonstrated a significant indirect effect of the feeling of emptiness on self-harm.

The majority of Thais are Buddhists, and practices such as meditation, adherence to the Five Precepts, and equanimity are integral to Buddhist teachings. The study’s findings suggest that targeting these inner strengths as protective factors could help diminish the impact of emotional emptiness on self-harm, offering a valuable strategy for developing interventions aimed at reducing self-harm in individuals with BPD symptoms. The Five Precepts are essential practices alongside meditation. Strategies related to the Five Precepts, meditation, and equanimity can be applied through social media applications. In Thailand, where the prevalence of BPD among Thai students is high, the population in this study, with an age range of 20 to 50 years, is particularly relevant. Individuals in this age range use smartphones regularly, from adolescence to adulthood. There is evidence that Thailand has a higher rate of smartphone adoption and a greater prevalence of internet and social media addiction (e.g., Facebook, Instagram) compared to most Asian countries [46]. A systematic review has shown that digital devices have become fundamental tools in daily life. The results demonstrate a positive relationship between the use of mobile health applications and topics such as sexual and reproductive health, prevention of harmful habits, and coping strategies for stress and difficult situations, as well as skills to improve depressive symptoms, self-esteem, and quality of life. Mobile health applications also promote the reduction of alcohol consumption [47]. We propose that new technologies can be used to implement the Five Precepts, such as through education on preventing sexual misconduct or alcohol consumption. Additionally, applications can be developed or innovated to support meditation and equanimity practices.

### 4.1. Implication of This Study and Future Research

Inner strength—encompassing the Five Precepts, meditation, and equanimity—that can help prevent self-harm may be developed through various methods. Often, patients learn to cultivate these strengths daily, with equanimity being promoted in individuals with BPD. The Five Precepts and equanimity (a part of the Four Immeasurables) are in Thailand common practices and along with meditation considered methods to enhance mental well-being.

In a clinical setting, these activities can be suggested. Patients with BPD symptoms may be encouraged to engage in meditation practices and follow the Five Precepts (which include abstaining from killing, stealing, sexual misconduct, lying, and the consumption of alcohol) as part of supplementary psychotherapeutic interventions.

Future research should focus on individuals with BPD in different cultures, as inner strength is considered a universal concept. More robust research should investigate whether providing training in equanimity, the Five Precepts, and meditation to patients with BPD symptoms, particularly those experiencing feelings of emptiness, can help prevent self-harm behavior.

### 4.2. Strengths and Limitation

To the best of our knowledge, this study is one of the first to demonstrate the beneficial role of the Five Precepts, meditation, and equanimity in relation to self-harm among individuals with BPD symptoms experiencing feelings of emptiness. However, several limitations should be addressed. Firstly, the BPD-symptom population was categorized using the SCID-II Personality Disorder (Borderline PD) rather than clinical diagnoses, which may lead to false-positive cases. Secondly, social desirability bias in self-reports may be unavoidable, and, thus, the interpretation of the results should be cautiously approached. Thirdly, our data cannot exclude participants who are currently receiving psychological treatment, which may have influenced reductions in self-harm and increases in inner strength due to therapy. Fourthly, this study was conducted in Thailand, and cultural factors might have influenced the outcomes, particularly those related to inner strength. Therefore, replication studies in other countries are warranted. Finally, due to the retrospective cross-sectional design of this study, causal inferences cannot be confirmed; a longitudinal design would be more appropriate for establishing causality. Conducting randomized controlled trials (RCTs) with pre-test and post-test assessments would provide valuable insights into the effectiveness of interventions, such as those related to the Five Precepts, meditation, and equanimity, in reducing self-harm and improving emotional regulation among individuals with BPD symptoms. This approach would allow for a more rigorous evaluation of the causal relationships between these inner strengths and the outcomes. Future studies could explore this design to further validate our retrospective study’s findings.

## 5. Conclusions

The present study has demonstrated a positive correlation between feelings of emptiness and self-harm. Conversely, inner strength—encompassing the Five Precepts, meditation, and equanimity—significantly mediates the relationship between feelings of emptiness in individuals with borderline personality disorder symptoms (BPD symptoms) and self-harm, showing a negative association. This novel evidence guides helping individuals with BPD symptoms who experience feelings of emptiness manage self-harm more effectively.

## Figures and Tables

**Table 1:** Sociodemographic data.

Variables	All (*n* = 302)	Self-Harm Group	Non-Self-Harm Group	Test Difference
Age (year)	36.56 ± 17.17	34.92 ± 17.10	41.35 ± 16.60	t (296) = −2.85, *p* = 0.005
Sex, female	195 (65.4%)	146 (65.8%)	49 (64.5%)	χ^2^ (1) = 0.042, *p* = 0.838
Education Lower Bachelor or higher	135 (45.3%) 163 (54.7%)	99 (44.6%) 123 (55.4%)	36 (47.4%) 40 (52.6%)	χ^2^ (1) = 0.176, *p* = 0.675
Marital status Together Alone	120 (40.3%) 178 (59.7%)	91 (41%) 131 (59%)	29 (38.2%) 47 (61.8%)	χ^2^ (1) = 0.189, *p* = 0.664
Feelings of emptiness	242 (80.1%)	212 (93.8%)	30 (23.3%)	χ^2^ (1) = 105.45, *p* < 0.001
OI-Depression ^1^	13.46 ± 5.37	14.24 ± 5.41	11.14 ±4.54	t (300) = 4.48, *p* < 0.001

^1^ OI-Depression = Outcome Inventory-Depression.

**Table 2:** Correlation coefficients between feelings of emptiness, self-harm, OI-Depression, the Five Precepts, meditation, and equanimity.

Variables	N(%) or Mean SD	Emptiness	Self-Harm	OI-Depression ^1^	Precepts	Meditation	Equanimity
Feelings of emptiness	242 (80.1%)	.					
Self-harm	226 (74.8%)	0.591 **	.				
OI-Depression ^1^	13.46 ± 5.4	0.309 **	0.251 **	.			
Precepts	2.83 ± 1.2	−0.236 **	−0.240 **	−0.081	.		
Meditation	1.72 ± 1.0	−0.250 **	−0.240 **	−0.162 **	0.420 **	.	
Equanimity	2.90 ± 1.2	−0.188 **	−0.232 **	−0.380 **	0.096	0.158 **	.

^1^ OI-Depression = Outcome Inventory-Depression, ** *p* < 0.01.

**Table 3:** Regression model of feelings of emptiness predicting self-harm.

Antecedent	Coeff. ^1^	95% CI	*p*-Value
X (emptiness)	0.565	0.507, 0.721	<0.001
Age	0.002	−0.002, 0.003	0.640
OI-Depression ^2^	0.080	−0.002, 0.015	0.118
R^2^ = 0.35			

^1^ Coeff. = Standardized coefficient, ^2^ OI-Depression = Outcome Inventory-Depression.

**Table 4:** The direct and indirect effects of feelings of emptiness, the Five Precepts, meditation, and equanimity on self-harm.

Antecedent	Coeff. ^1^ (95% CI), *p*-Value			
	M1 (Precepts)	M2 (Meditation)	M3 (Equanimity)	Y (Self-Harm)
X (feelings of emptiness)	−0.200 (−0.319, −0.073), 0.001	−0.164 (−0.303, 0.028), 0.018	−0.071(−0.182, 0.039), 0.210	0.534, (0.417, 0.647), <0.001
X (feelings of emptiness) *				0.034 (0.009, 0.075), 0.005
Precept				−0.085 (−0.179, 0.009), 0.077
Meditation				−0.060 (−0.179, 0.055), 0.304
Equanimity				−0.103 (−0.194, −0.017), 0.016
	R^2^ = 0.09	R^2^ = 0.17	R^2^ = 0.15	R^2^ = 0.38

^1^ Coeff. = Standardized coefficient, * Indirect effect.

## Data Availability

The datasets used and/or analyzed during the current study are available from the corresponding author upon reasonable request.
